# Awareness Level Regarding Brain Death and the Acceptance of Organ Donation in Eastern Province, Saudi Arabia

**DOI:** 10.7759/cureus.37760

**Published:** 2023-04-18

**Authors:** Jinan M Aljasem, Abdullah H Bohamad, Abdulaziz Y Alahmed, Hadeel H Buali, Ali H Alhussain, Mohammed Aldawood, Ali M Aljasem, Sherif M Saleh

**Affiliations:** 1 Medical School, King Faisal University, Al Ahsa, SAU; 2 Obstetrics and Gynaecology, King Faisal University, Al Ahsa, SAU; 3 General Surgery, King Faisal University, Al Ahsa, SAU

**Keywords:** awareness, brain death, brain death organ management, organ transplant, organ donor shortage, transplantation

## Abstract

Background

There is a worldwide shortage of organ donations. In the United States, 20% of people on transplant waiting lists pass away annually due to the lack of accessible organs. Patients with brain death can donate organs, which may save other patients’ lives. The Saudi Ministry of Health endorses brain death as equivocal to whole-body death. A study conducted in Saudi Arabia showed that there was a mild to moderate level of awareness regarding brain death. This study aimed to investigate the awareness and knowledge level regarding brain death and the acceptance of organ donation among the general population in Eastern Province, Saudi Arabia.

Methodology

An observational, cross-sectional study was conducted among 1,740 adults using an online questionnaire created and published in February 2023 to collect data from Saudi males and females aged 18 or older who were willing to participate in the study. The data were analyzed using SPSS version 23.0 (IBM Corp., Armonk, NY, USA) after collecting and entering them using the Windows version of Microsoft Office Excel 2016.

Results

Overall, 85.6% of the study participants had heard about organ donation. Of them, about 42.4% were aware of brain death. Further, 40% of participants were in agreement with organ donation. According to the findings, the majority of participants (60.9%) believed that a person could donate his or her organs during their life, while only 42.6% were unaware that they could donate their organs during death. Only 10.8% of participants knew that blood can be donated. There was no significant association between factors associated with organ donation and gender, education level, or monthly income.

Conclusions

This study concluded that study participants had a low level of awareness about brain death. Understanding brain death is essential for persuading people to donate their organs. Thus, more has to be done to inform and educate people about brain death and how it affects organ donation.

## Introduction

According to the American Academy of Neurology (AAN) and the British National Health Service, any patient who has had an irreversible loss of all brain functions, including brainstem function loss, by showing loss of respiratory function (apnea), absence of brainstem reflexes, and total loss of consciousness (coma), is considered to have brain death [1,2]. The Saudi Ministry of Health has settled the issue of brain death and judged that it is equivocal to body death. This was done based on medical and religious Islamic backgrounds. Moreover, the components of brain death are the same as in the British and American definitions, except that the Islamic Sharia insists on treating reversible brain insults such as metabolic diseases or drug intoxication due to the significance of soul preservation. Furthermore, it is mandatory to have two consultant examiners to diagnose brain death [3]. There are a variety of causes leading to brain death that include stroke, subarachnoid hemorrhage, intracerebral hemorrhage, and cardiopulmonary arrest with adequate resuscitation [4]. In the United States, brain death accounts for 2% of all adults and 5% of all pediatric mortality in hospitals [5]. Unfortunately, brain death is an irreversible condition. However, patients with brain death can donate organs, which may save other patients’ lives [6]. The main indication of organ transplantation is end-stage organ dysfunction [7]. There is a worldwide shortage of organ donations. Annually, the paucity of available organs results in the death of about 20% of individuals on transplant waiting lists [8,9]. In 2021, patients who were on the nation’s transplant waiting list accounted for 105,800. Moreover, 90% of adults in the United States support organ donation, and 169 million people have signed up to be donors [10]. According to data from the Saudi Center for Organ Transplantation, there have been 11,866 incidents of brain death since the inception of organ donation in 1986 until 2016 [11]. The main concern with organ donation is obtaining the patient’s and family’s consent before the transplant. A study conducted in India found that although people were generally unaware of the concept of brain death, they were aware of organ donation [12]. Another study conducted in Saudi Arabia showed that there was a mild-to-moderate level of awareness regarding brain death [13]. This study aimed to investigate the awareness and knowledge level regarding brain death and the acceptance of organ donation among the general population in Eastern Province, Saudi Arabia.

## Materials and methods

Study design and selection criteria

This study was designed as a community-based, cross-sectional study to assess the general population’s awareness and knowledge of brain death and acceptance of organ donation among Saudi citizens older than 18 years, willing to participate, and who completed the entire questionnaire by utilizing an online tool. On the other hand, participants who were non-Saudi citizens, those who declined to participate, and those who did not finish the entire questionnaire were excluded.

Study population and sample size

The population consisted of participants who fulfilled the inclusion and exclusion criteria and were involved in this study. A total of 1,701 respondents constituted the sample size, which was calculated using the Richard Geiger equation with a 95% confidence level and a 5% margin of error.

Questionnaire

A Google Forms questionnaire was translated into Arabic, which is the native language of Saudi Arabia, and distributed randomly on social media platforms such as WhatsApp, Telegram, Twitter, Facebook, and Instagram. The questionnaire consisted of three domains. The first domain was socioeconomic, which included four questions regarding gender, age, educational level, and monthly income. The second domain was a brain death knowledge assessment, which included 12 questions, of which eight questions assessed the knowledge level regarding the definition, causes, and prognosis of brain death, and four questions assessed the knowledge level regarding the source of information and the interest to learn more about brain death. The knowledge level was calculated by giving the correct answer a score of 1 and the wrong answer a score of 0. The mean for each question was then calculated, with scores ranging between 0 and 8. The third domain was about awareness and acceptance of organ donation and included nine questions.

Data management and statistical analysis

The data were analyzed using SPSS version 23.0 (IBM Corp., Armonk, NY, USA) after collecting and entering data using the Windows version of Microsoft Office Excel 2016. With a significance level of 0.05, the data from the questionnaire were analyzed using descriptive analysis and the chi-square test.

Ethical consideration

The approval was obtained from the Ethics Committee at King Faisal University of Medical Sciences. The purpose of the study was explained to all participants, and only those who provided informed consent were included in the study. All participants were guaranteed that their confidentiality would be maintained, and no personal information was requested on the questionnaire.

## Results

The results were obtained from 1,701 participants (55.6% female and 44.4% male), most of whom (58.5%) were aged 18-30 years, with 15.2% aged 31-40 years, 15.7% aged 41-50 years, and 10.6% aged over 50 years. Most participants (62.7%) had a university education, 24.6% had a secondary education, 8.6% were postgraduates, 2.4% had an intermediate education, and 1.8% had a primary education. The monthly income of the participants was as follows: 48.4% had less than 3,000 Saudi Riyals (SR), 25.7% had 3,000-10,000 SR, 18.5% had 10,000-20,000 SR, and 7.3% had more than 20,000 SR (see Table 1).

**Table 1:** Demographic characteristics. SR: Saudi Riyal

Variables	Categories	N	%
Gender	Male	756	44.4
	Female	945	55.6
Age	18–30 years	995	58.5
	31–40 years	258	15.2
	41–50 years	267	15.7
	Over 50 years	181	10.6
Education level	Primary	30	1.8
	Intermediate	40	2.4
	Secondary	418	24.6
	University	1,066	62.7
	Postgraduate	147	8.6
Monthly income	Less than 3,000 SR	824	48.4
	3,000–10,000 SR	438	25.7
	10,000–20,000 SR	314	18.5
	Over 20,000 SR	125	7.3

The results showed that the knowledge level of participants was 42.4%, with a mean of 3.69. The percentage of correct answers for each question was as follows: “Are you familiar with the term brain death?” (85.6%); “Is brain death different from a coma?” (75.8%); “Brain death is...” (72.6%); “Can brain death be cured?” (54.6%), “Do you think brain death is real death?” (37.4%); “What are the signs of brain death?” (25%); “Can brain-dead patients donate all their organs?” (17.4%); and “What do you think causes brain death?” (0.4%) (see Table 2).

**Table 2:** Knowledge about brain death.

Questions	Frequency (correct answer)	%
Are you familiar with the term brain death?	1,456	85.6
Brain death is…	1,323	72.6
What do you think causes brain death?	7	0.4
What are the signs of brain death?	425	25
Can brain-dead patients donate all their organs?	296	17.4
Can brain death be cured?	929	54.6
Do you think brain death is real death?	637	37.4
Is brain death different from a coma?	1,289	75.8
Knowledge level	3.69	42.4

The results showed that the most informative source on brain death for participants was friends and family (25.5%), followed, in descending order, by healthcare providers (23.6%), non-governmental web searches and other social media platforms (23%), publicly available educational programs (15.4%), official government platforms (7%), personal experience (3.2%), drama (1.5%), and, finally, universities (0.8%) (see Table 3).

**Table 3:** Information sources on brain death.

Sources	Frequency	%
Healthcare providers	344	23.6
Official government platforms	102	7
Friends and family	371	25.5
Publicly available educational programs	224	15.4
Non-governmental web searches and other social media platforms	335	23
University	11	0.8
Personal experience	47	3.2
Media	22	1.5

The results showed that 67.7% of the participants thought that they did not have enough information to understand and know about brain death, while 32.3% did. A total of 76% of the participants wanted more information about brain death, while 24% did not (see Table 4).

**Table 4:** Attitude toward new information on brain death.

Questions	Yes	No
Do you think you have enough information to understand and know about brain death?	550	1,151
	32.3%	67.7%
Do you want more information about brain death?	1,292	409
	76%	24%

The results showed that the information people wanted to learn about the most was how to prevent brain death (64.3%), followed by, in descending order, the fate of patients with brain death (62.1%), how to diagnose patients with brain death (58%), how brain death happens (53.9%), what is brain death (48.8%), causes of brain death (48.1%), treatment of brain death, all of the above (0.2%), whether it is permissible to donate organs, the difference between brain death and coma, and how to deal with a person with brain death (0.1%) (see Table 5).

**Table 5:** Issues about brain death you would like to know more about in the future.

Information	N	%
What is brain death?	631	48.8
Causes of brain death	621	48.1
How does brain death happen?	697	53.9
The fate of patients with brain death	802	62.1
How to diagnose patients with brain death?	749	58
How to prevent brain death?	831	64.3
Treatment of brain death	2	0.2
Is it permissible to donate organs?	1	0.1
The difference between brain death and coma	1	0.1
How to deal with a person with brain death?	1	0.1
All of the above	2	0.2

The results showed that there was a significant difference between males and females (t = −2.208, p = 0.027), between different ages (F = 15.212, p < 0.001), between education levels (F = 11.342, p < 0.001), and between monthly income (F = 3.919, p = 0.008) in the knowledge level of brain death (see Table 6).

**Table 6:** The difference in the level of knowledge due to demographic characteristics SR: Saudi Riyal; ANOVA: analysis of variance

Variables	Categories	Mean	Test	Statistics	P-value
Gender	Male	3.6	Independent-samples test	−2.208	0.027
	Female	3.75			
Age	18–30 years	3.88	ANOVA	15.212	0000
	31–40 years	3.45			
	41–50 years	3.39			
	Over 50 years	3.42			
Education level	Primary	2.53	ANOVA	11.342	0.000
	Intermediate	2.78			
	Secondary	3.59			
	University	3.78			
	Postgraduate	3.79			
Monthly income of knowledge	Less than 3,000 SR	3.78	ANOVA	3.919	0.008
	3,000–10,000 SR	3.51			
	10,000–20,000 SR	3.64			
	Over 20,000 SR	3.83			

The results showed that most of the participants (60.9%) thought that a person can donate his/her organs during their lifetime, 56.7% could donate after death, and 42.6% could donate after being announced brain dead (see Table 7).

**Table 7:** When do you think a person can donate his/her organs?

Time	N	%
After announcing brain death	660	42.6
During lifetime	942	60.9
After death	878	56.7

The results showed that the most-donated organ was the kidney (78.1%), followed by the liver (73%), eye (65.1%), lung (44.2%), and heart (10.8%) (see Table 8).

**Table 8:** Organs that can be donated.

Organs	N	%
Kidney	1,317	78.1
Liver	1,231	73
Heart	182	10.8
Eye	1,099	65.1
Lung	745	44.2

The results showed that most participants (68.9%) knew that there are official centers for organ donation in the Kingdom of Saudi Arabia, while 31.1% did not. Of those who did know, only 42.2% were aware of the laws and regulations for organ donation in cases of organ transplant and brain death, while 57.8% were not. Most of the participants (40%) agreed with organ donation, 31.8% were neutral, 17.5% were unsure, and only 0.6% disagreed with it. It was shown that 73.4% of participants would donate their organs if religion encouraged organ donation, while 26.6% would not. Most participants (48.8%) would donate their organs after death, 41.1% saw no difference between donating during their lifetime or after their death, 10.1% would donate during their lifetime only, 69.4% wanted to donate their organs to either relatives or non-relatives, 17% wanted to donate only to relatives, and 2.9% wanted to donate only to non-relatives (see Table 9).

**Table 9:** Perception of organ donation.

Questions	Categories	N	%
Did you know that there are official centers for organ donation in the Kingdom of Saudi Arabia?	Yes	1,172	68.9
	No	529	31.1
If your answer to the previous question was “yes,” are you aware of the laws and regulations for organ donation in cases of organ transplant and cases of brain death?	Yes	494	42.2
	No	678	57.8
What is your position on organ donation?	I disagree with it	181	10.6
	I’m not sure	298	17.5
	I am neutral	541	31.8
	I agree with it	681	40
If religion encouraged organ donation, would you donate your organs?	Yes	1,249	73.4
	No	452	26.6
When can you donate your organs if you are willing to do so?	I see no difference in donating during my lifetime or after death	624	41.1
	After my death	742	48.8
	During my lifetime only	154	10.1
If you want to donate your organs, to whom do you want to donate them?	To both relatives and non-relatives	1,181	69.4
	Only to relatives	290	17
	Only to non-relatives	49	2.9

The results showed that the highest motivating factor for organ donation was the desire to help others (70.5%), followed by religious motives (66.7%), the health status of the recipient (40.9%), social motives (37.7%), the age of the recipient (17%), and financial motives (4.5%) (see Table 10).

**Table 10:** Motives for organ donation.

Motives	N	%
Religious motives	1,014	66.7
The desire to help others	1,072	70.5
The health status of the recipient	621	40.9
Social motives	573	37.7
Financial motives	69	4.5
Age of the recipient	259	17

The results showed that there was a significant association between age and organ donation (chi-square = 13.353, p = 0.0004); a total of 44.7% of participants aged 18-30 years would donate their organs, while 13.8% would not donate; 10.9% aged 31-40 years would donate, while 4.2% would not; 10.5% aged 41-50 years would donate, while 5.2% would not; and 7.2% aged over 50 years would donate, while 3.4% would not. There was no significant association with other factors (see Table 11).

**Table 11:** Factors associated with organ donation. SR: Saudi Riyal

Variables	Categories	Would you donate your organs?			Chi-square	P-value
			Yes	No		
Gender	Male	N	550	206	0.319	0.572
		%	32.30%	12.10%		
	Female	N	699	246		
		%	41.10%	14.50%		
Age	18–30 years	N	761	234	13.353	0.004
		%	44.70%	13.80%		
	31–40 years	N	186	72		
		%	10.90%	4.20%		
	41–50 years	N	179	88		
		%	10.50%	5.20%		
	Over 50 years	N	123	58		
		%	7.20%	3.40%		
Education level	Primary	N	21	9	4.259	0.372
		%	1.20%	0.50%		
	Intermediate	N	24	16		
		%	1.40%	0.90%		
	Secondary	N	305	113		
		%	17.90%	6.60%		
	University	N	789	277		
		%	46.40%	16.30%		
	Postgraduate	N	110	37		
		%	6.50%	2.20%		
Monthly income	Less than 3,000 SR	N	617	207	5.725	0.126
		%	36.30%	12.20%		
	3,000–10,000 SR	N	317	121		
		%	18.60%	7.10%		
	10,000–20,000 SR	N	217	97		
		%	12.80%	5.70%		
	Over 20,000 SR	N	98	27		
		%	5.80%	1.60%		

## Discussion

This study was conducted to investigate the awareness and knowledge of brain death and the acceptance of organ donation among the general population in the Eastern Province of Saudi Arabia. We observed a moderate level of knowledge on brain death of almost 42%, which corresponds with the results of a previous study conducted in Saudi Arabia in which a moderate level of knowledge regarding brain death was found [13]. This could be explained by the limited background of health information, especially brain death-related information, among the Saudi population. In this study, the most common source of information (25%) on brain death was family and friends, followed by social media (23%). Another study conducted in Saudi Arabia reported that the main source of information regarding brain death was social media [14]. This finding partially agrees with ours because the general population usually depends on social media as the main source of information; however, due to our small sample size, this finding might be slightly underestimated.

The public in the Kingdom of Saudi Arabia lacks an understanding of brain death, which can negatively impact organ donation. This study aimed to evaluate the current level of knowledge regarding brain death among the population. Previous studies have not concentrated on awareness of brain death but have instead focused on knowledge, attitudes, and practices related to organ donation. In addition, no validated Arabic questionnaire on brain death was available for use in this study. Hence, we developed a questionnaire based on validated evaluations, which was later translated from English to Arabic.

Our findings suggest similar means of knowledge levels for both males and females (3.6 and 3.75, respectively). The results indicate that the general population in the Kingdom of Saudi Arabia possesses limited-to-moderate knowledge of brain death. This is consistent with previous findings, where less than half of the participants surveyed demonstrated an adequate understanding of brain death and organ donation [15]. However, this finding differs from the results of two studies conducted in Turkey and Iran that indicated inadequate awareness and a necessity to enhance the understanding of brain death and organ donation [16,17]. Furthermore, numerous studies have investigated the awareness of organ donation and brain death among various groups in the Kingdom of Saudi Arabia, including university and secondary school students in the southern and Al-Ahsa regions [18-20]. These studies have revealed an insufficient understanding of brain death and underscored the significance of raising awareness on the topic to enhance knowledge among the Saudi population.

Our findings revealed that 48.8% of participants were familiar with the notion of brain death, surpassing the results of Febrero et al., whose study indicated that only 38% comprehended the concept of brain death. However, this is lower than the values reported in the Al-Ahsa study at 84.6% [18], and in the study by Al-Qahtani et al. at 76.9% [13]. We observed that the mean knowledge score on brain death increased significantly with the level of education, where those with a postgraduate degree had a score of 3.79 compared with those with primary education at just 2.53. Individuals with a higher level of education are more likely to have encountered or studied the concept of brain death and thus may not require additional information on the subject. No significant correlations were found between knowledge scores and other sociodemographic factors, such as gender. The limitations of this study are its small sample size and its single geographic location.

Recommendation

Future studies should aim to enroll participants from various regions of the country in equal numbers to obtain more precise results that can be generalized across the entire population. In summary, our findings indicate that the general population of the Kingdom of Saudi Arabia possesses limited-to-moderate knowledge about brain death. To enhance awareness of this topic, we recommend using various mass media platforms. Additionally, education and awareness programs on brain death are necessary, especially concerning prevention and causes, in both schools and workplaces.

## Conclusions

This study showed that the overall awareness level regarding brain death was suboptimal. There was a significant association between age and organ donation, but there was no such association with gender, education level, or monthly income. Most participants wanted more information on brain death, and most participants agreed with organ donation; however, the majority were unaware that they can donate an organ while they are brain dead. Few participants were aware that hearts can be transplanted. The authors suggest launching additional awareness campaigns about brain death and organ donation. More studies are needed to determine the causes of people’s reluctance to donate their organs.

## Appendices

Socio-demographical data:

Gender:

Male

Female

Age:

18-30 years

31-40 years

41-50 years

Older than 50 years

Educational level:

Primary school

Intermediate school

High school

University education

Income average:

<3,000 SR/month

3,000-10,000 SR/month

10,000-20,000 SR/month

>20,000 SR/month

Brain death

Are you familiar with the term brain death?

Yes

No

If yes, what is your information source?

Official government platforms

Non-governmental web search and other social media

Healthcare provider

Friends and family

Personal experience

Publicly available educational programs

Brain death is...

Stopping of heart 

Reversible stopping in brain function 

Irreversible stopping in brain function

What do you think causes brain death?  

Stroke

Road traffic accident

Heart attack

Tumors

What are the signs of brain death? (choose more than one answer)?

Patient does not respond to light*

Patient does not show any reaction to pain*

Eyes do not move when the head is moved*

Patient does not breathe without a ventilator*

Patient can respond to light

Patient shows reaction to pain

Patient can breathe without a ventilator

I don’t know

All the above

Can brain-dead patients donate all their organs?

Yes 

No

I don’t know

Can brain death be cured?

Yes

No

I don’t know

Do you think brain death is real death?

Yes

No

I don’t know

Is brain death different from a coma?

Yes

No

I don’t know

Do you think you have enough information to understand and know brain death?

Yes

No

Do you want more information about brain death?

Yes

No

What would you like to know more about brain death in the future?

Other

What is the brain death

How to diagnose them

The fate of brain death patients

How does brain death happen

How to prevent brain death

Causes

When do you think a person can donate his/her organs? (More than 1 answer can be chosen)

During lifetime

After announcing brain death

After the death

I don't know

Which of the following organs do you think can be donated?

Kidneys

Liver

Heart

Eyes

Lungs

I don’t know

Are you aware of the presence of centers and official agencies for organ donation in Saudi Arabia?

Yes

No

If you answered the previous question with yes, are you aware of the laws and regulations related to organ donation, brain death, and organ transplantation in Saudi Arabia?

Yes

No

What is your attitude/perception of organ donation?

In agreement with it

In disagreement with it

I’m not sure

Neutral

When would you prefer to donate your organs if you agree to do so and are willing to do so?

During my lifetime only

After my death

I see no difference in donating during my lifetime or after death

If you are willing to donate your organs, to whom are you willing to donate your organs?

Only to relatives

Only to non-relatives

To both relatives and non-relatives

If you are willing to donate your organs, what are your motives?

Religious motives

Social motives

The desire to help others

Age of the receivers

The health status of the receiver

Financial motives

If laws and religion encourage donation, will you donate?

Yes

No
